# The power of creatine plus resistance training for healthy aging: enhancing physical vitality and cognitive function

**DOI:** 10.3389/fphys.2024.1496544

**Published:** 2024-12-03

**Authors:** Diego A. Bonilla, Jeffrey R. Stout, Darren G. Candow, José Daniel Jiménez-García, Luis M. Gómez-Miranda, Melinna Ortiz-Ortiz, Scott C. Forbes, Sergej M. Ostojic, Salvador Vargas-Molina, Richard B. Kreider

**Affiliations:** ^1^ Research Division, Dynamical Business and Science Society–DBSS International SAS, Bogotá, Colombia; ^2^ Research Group in Physical Activity, Sports and Health Sciences (GICAFS), Universidad de Córdoba, Montería, Colombia; ^3^ Hologenomiks Research Group, Department of Genetics, Physical Anthropology and Animal Physiology, University of the Basque Country (UPV/EHU), Leioa, Spain; ^4^ Physiology of Work and Exercise Response (POWER) Laboratory, Institute of Exercise Physiology and Rehabilitation Science, University of Central Florida, Orlando, FL, United States; ^5^ Faculty of Kinesiology and Health Studies, University of Regina, Regina, SK, Canada; ^6^ Department of Health Sciences, Faculty of Health Sciences, University of Jaén, Jaén, Spain; ^7^ Facultad de Deportes, Universidad Autónoma de Baja California, Tijuana, Mexico; ^8^ Facultad de Medicina y Psicología, Universidad Autónoma de Baja California, Tijuana, Mexico; ^9^ Department of Physical Education Studies, Brandon University, Brandon, MB, Canada; ^10^ Applied Bioenergetics Lab, Faculty of Sport and Physical Education, University of Novi Sad, Novi Sad, Serbia; ^11^ Department of Nutrition and Public Health, University of Agder, Kristiansand, Norway; ^12^ Faculty of Health Sciences, University of Pecs, Pécs, Hungary; ^13^ Physical Education and Sport, Faculty of Medicine, University of Málaga, Málaga, Spain; ^14^ Exercise and Sport Nutrition Laboratory, Human Clinical Research Facility, Texas A&M University, College Station, TX, United States

**Keywords:** creatine monohydrate, sarcopenia, nutrition therapy, elder nutritional physiology, resistance training, skeletal muscle, creatine

## Introduction

Sarcopenia is generally defined as an age-related musculoskeletal disease characterized by the reductions in muscle strength, lean/muscle mass (quality) and functional ability leading to various adverse health outcomes, including impaired physical function and reduced quality of life (Kirk et al., 2024). Sarcopenia is also associated with cognitive decline in older adults (Arosio et al., 2023). As sarcopenia is a potentially reversible condition whose prevalence increases with age, several non-pharmacological strategies have been developed to counteract its progression in older adults (Cannataro et al., 2021; Cannataro et al., 2022).

Sedentary and physically inactive older adults exhibit a reduced myofibrillar protein-synthesis response to dietary protein intake, which significantly accelerates the progression of sarcopenia (Moore, 2014). This issue is further exacerbated in obese elderly populations. Age-related muscle ‘anabolic resistance’ becomes particularly pronounced in response to low or moderate protein intake, a dietary pattern commonly observed in older individuals (Aragon et al., 2023). It is now recognized that a daily intake of at least 1.0 g of protein per kg/day, rich in essential amino acids (primarily leucine) is crucial to maintaining positive protein balance in musculoskeletal tissue. While we acknowledge the importance of high protein intake in older adults, we will not elaborate on this topic, given the extensive international and governmental efforts already in place. For example, the ESPEN Expert Group (Deutz et al., 2014) and the PROT-AGE Study Group (Bauer et al., 2013) provide international guidelines, while governmental initiatives such as the World Health Organization’s ‘Keep fit for life: meeting the nutritional needs of older persons’ (Organization WH and Policy TU-SoNS, 2002) and the European Commission’s ‘Dietary recommendations for protein intake for adults and older adults’ (Commission) address this issue comprehensively.

In this opinion article, we advocate for the combination of creatine monohydrate supplementation and resistance training as a safe and effective non-pharmacological strategy to prevent and treat sarcopenia that should be internationally recognized by health practitioners and public health organizations.

### Creatine is a conditionally essential nutrient for lifelong vitality

Creatine is derived from reactions involving amino acids in the liver and brain. Approximately 95% of the creatine pool in the body is found in skeletal muscle, while 5% is found in other tissues with high energy demands such as cardiomyocytes, hepatocytes, kidney cells, inner ear cells, enterocytes, spermatozoa, and photoreceptor cells. Creatine in its phosphorylated form, phosphocreatine (PCr), plays a pivotal role in sustaining adenosine triphosphate (ATP) in these cells (Bonilla et al., 2021a).

Creatine is naturally present in meat, fish, and poultry with an estimated daily requirement of approximately 2 g·day^−1^ for a 70-kg male to maintain normal creatine levels in the human body (Kreider and Stout, 2021; Wallimann et al., 2011). Notwithstanding, research across diverse populations has demonstrated that endogenous creatine synthesis may be insufficient under numerous physiological and pathological conditions (Ostojic and Forbes, 2022).

Following the groundbreaking research of Dr. Roger Harris in 1992 (Harris et al., 1992), creatine monohydrate became widely available as a dietary supplement in the United States and Europe, fully compliant with the U.S. Food and Drug Administration (FDA) regulations. Given its natural presence in food and its availability as a supplement before 15 October 1994, creatine was grandfathered under the Dietary Supplement Health and Education Act (DSHEA) as a legal dietary supplement in the U.S. Recently, the FDA expressed no objections to a Generally Recognized as Safe (GRAS) application, permitting the inclusion of creatine monohydrate as a food additive in various food products (GRAS Notice 931) (USFaD and Inventories, 2020). Further, after consultation on novel food status under Regulation (EU) 2015/2283, creatine is not considered a novel food as creatine monohydrate has been used for human consumption to a significant degree in the European Union before 15 May 1997 (Safety EHaF, 2023). Creatine is approved for inclusion in dietary supplements and/or food products in numerous countries, including Canada, Australia, the European Union, Japan, South Korea, and Brazil, among others (Jager et al., 2011). Extensive research, including randomized, double-blind, placebo-controlled clinical trials, have consistently demonstrated that creatine monohydrate supplementation is both safe and effective in humans (Kreider and Stout, 2021; Kreider et al., 2017; Kreider et al., 2022), including older adults (Candow et al., 2022; Candow and Moriarty, 2024). Thus, there is a strong consensus within the scientific community that creatine monohydrate supplementation (e.g., 20 g/day or 0.3 g/kg/day for 5–7 days; 3–5 g/day or 0.03 g/kg/day; or 0.1–0.14 g/kg/day) can safely and effectively enhance exercise performance capacity and training adaptations in both untrained and trained individuals, regardless of exercise interventions, biological sex or age (Kreider and Stout, 2021; Kreider et al., 2017; Burke et al., 2019; Candow et al., 2019; Forbes et al., 2021a; Kley et al., 2013; Maughan et al., 2018; Bakian et al., 2020; Korovljev et al., 2021a; Korovljev et al., 2021b; Korovljev et al., 2021c; Ostojic, 2021a; Ostojic, 2021b; Ostojic, 2021c; Ostojic et al., 2021a; Todorovic et al., 2023; Ostojic et al., 2024a; Ostojic et al., 2024b).

Consequently, an adequate intake of dietary creatine is likely conditionally essential for maintaining optimal health and promoting growth (Ostojic and Forbes, 2022). Due to the energy and mechanical optimization of cells (Bonilla et al., 2021a), increasing intracellular creatine levels through nutritional supplementation reduces protein degradation (Parise et al., 1985), promotes activation of satellite cells (Olsen et al., 2006), and increases whole-body lean tissue mass (Pashayee-Khamene et al., 2024). The cellular bioenergetics improvements after creatine supplementation offer potential benefits beyond musculoskeletal tissue, such as in the brain, the heart, vascular health, immune system, among others (Roschel et al., 2021; Clarke et al., 2021; Solis et al., 2021; Balestrino, 2021), especially in older adults (Candow et al., 2019; Gielen et al., 2021). In the context of this opinion article, several studies show that creatine monohydrate supplementation can augment muscle mass, physical and cognitive function in older adults (Kreider et al., 2017; Candow et al., 2021; Forbes et al., 2021b), with more clinically significant effects when combined with resistance training (Delpino et al., 2022).

### Resistance training offsets sarcopenia

Strength training involves contracting skeletal muscles to work against an external force. Resistance training, a form of strength training, involves different types of applied external forces, such as body weight, free weights, or resistance bands. In this article, we focus on resistance training, emphasizing its role in increasing muscle mass and strength, which are linked to improved health outcomes. Resistance training offers several physical benefits, including enhanced muscle strength, endurance, and power, along with increased bone mineral density and connective tissue remodeling. Clinically, it contributes to cardiometabolic health, helps prevent neurodegenerative disorders and mental health issues, and improves functionality, leading to reduced frailty and a lower risk of falls (American College of Sports et al., 2009; Cunha et al., 2024). Based on the available evidence, resistance training constitutes a key component of physical conditioning programs aimed at improving activities of daily living, self-care, and quality of life while reducing all-cause mortality in older adults (Cannataro et al., 2022; Kraschnewski et al., 2016).

The resistance exercise recommendations provided by the American College of Sports Medicine (ACSM) in collaboration with the American Heart Association (AHA) emphasize a training frequency of at least 2 days per week. The recommended intensity ranges from moderate (Moore, 2014; Aragon et al., 2023) to vigorous (Deutz et al., 2014; Bauer et al., 2013) on a scale of 0–10. The program should include progressive weight training with 8–10 exercises targeting major muscle groups, performing 8–12 repetitions per exercise (American College of Sports et al., 2009). Additionally, the National Strength and Conditioning Association (NSCA) suggests a resistance training regimen for older adults that involves 8–10 different free-weight or machine-based exercises, focusing on multi-joint movements and power/explosive training. This regimen includes 1-3 sets per exercise per muscle group, with 8–12 or 10–15 repetitions at 70%–85% of 1-RM, performed 2–3 days per week (per muscle group) (Fragala et al., 2019). Recent clinical evidence indicates that both low and high-frequency resistance training effectively enhances muscular strength, skeletal muscle mass, and muscle quality in older women with sarcopenia (Dos Santos et al., 2024). Cluster-set resistance training is an alternative approach that warrants further investigation in older adults, given its potential effectiveness in achieving superior results with less overall effort (Cannataro et al., 2022).

Before starting a resistance training program for older adults, it is advisable to use the ACSM’s exercise preparticipation screening tool for risk stratification (available at the ACSM Resource Library) and to establish goals based on a periodization plan. Assessing perceived exertion and pain levels are well-established methods for monitoring internal load and pain thresholds in different populations, including those at risk of frailty like older adults (Izquierdo et al., 2021). These straightforward techniques are effective for accurately tracking training intensity and making necessary adjustments (Falk Neto et al., 2020; Yu et al., 2021). Our recent findings suggest that using rating of perceived exertion (RPE) scales and perceived movement velocity is a valid, cost-effective, and practical method for assessing resistance training load progression and complementing other training metrics. Exercise professionals should ensure that participants are familiar with RPE scales and consider factors that may influence perceived exertion, such as training status, motivation, and environmental conditions (Petro et al., 2024).

We have previously outlined a comprehensive framework for understanding the etiology of resistance training-related injuries and provided detailed recommendations for exercise professionals, clinical exercise physiologists, and health practitioners to consider when designing exercise programs for older adults (Bonilla et al., 2022). Accordingly, it is essential to include sessions dedicated to familiarizing individuals with proper exercise techniques, irrespective of their experience or the type of resistance used (e.g., free weights, machine-based, or bodyweight exercises). These sessions should focus on teaching, observing, and correcting exercise techniques. Adhering to the safety principle in resistance training, it is crucial to select exercises that do not impact participants’ wellbeing. It is advisable to begin with simpler exercises (using machines or light weights) and gradually progress to more complex exercises (such as free weights with moderate-to-high loads) that demand greater control.

To enhance the health, functional capacity, and quality of life for older adults, it is recommended to implement community-based exercise programs that focus on moderate-intensity activities, such as circuit resistance training. Meta-analytic evidence demonstrates that these programs are more effective than standard care in improving functional capacity and health-related quality of life (Desveaux et al., 2014). Social media interventions targeting lifestyle behaviors—such as online communities, interactive web platforms, online education, and healthcare resources—have been well-received and can effectively promote physical activity among older adults (McKeon et al., 2022). Furthermore, promoting exercise programs that incorporate prescribed home-based therapy is also beneficial (Ricke et al., 2023). A notable example of these applications is the Curves Women’s Health and Fitness Initiative at the Exercise and Sport Nutrition Laboratory (Texas A&M University). This initiative has highlighted several benefits of circuit-style resistance training programs and the use of technology for health applications in women living with aging conditions (e.g., type-2 diabetes, obesity, etc.), including older females (Kerksick et al., 2009; Kreider et al., 2011; Magrans-Courtney et al., 2011; Galbreath et al., 2018; Kerksick et al., 2020).

Finally, creating and sustaining active environments that offer safe and equitable access to resistance exercise opportunities is crucial for older adults. This involves ensuring that cities and communities provide spaces and facilities that accommodate individuals of all ages and abilities, facilitating regular engagement in resistance training. For recommendations detailing the characteristics of resistance training protocols prescribed for older adults and a description of program variables, readers should refer to the Position Statement from the National Strength and Conditioning Association (Fragala et al., 2019) and the recent scoping review on the topic by da Silva et al. (2023).

## The benefits of creatine supplementation plus resistance training in older adults

Creatine monohydrate supplementation (≥5 g/day; 0.1–0.14 g/kg/day) during a resistance training program has the potential to preserve mental and physical abilities and mitigate sarcopenia and its associated risks. Table 1 shows large-scale epidemiological, meta-analytic and regulatory evidence supporting the importance of creatine intake and its positive effects when combined with resistance training to enhance cognitive function and physical vitality in older adults.

**TABLE 1:** Epidemiological, meta-analytic and regulatory evidence supporting the intake of creatine alone or combined with resistance training in older populations.

Cross-sectional epidemiological studies		
Study characteristics	Main Findings	Reference
NHANES 2005–2012. *n* = 22,692 both men and women aged 20±85 years	The study’s findings suggest a link between dietary creatine and depression. Authors reported that “*depression prevalence was 42% higher among adults in the lowest quartile (0–0.26 g) compared to adults in the highest quartile (0.70–3.16 g) of creatine consumption*.”	Bakian et al. (2020)
NHANES 2001–2002. *n* = 1,340 older adults (F: 51.8%) aged 71.4 [7.8] years	A significant positive correlation was observed between cognitive function and creatine intake across the entire sample. Participants who consumed more than 0.95 g of creatine per day exhibited higher scores on cognitive functioning assessments compared to those with lower creatine intake (*p* < 0.05). This indicates that dietary creatine may offer protective benefits against diminished cognitive performance in the older population	Ostojic et al. (2021)
NHANES 2017–2018. *n* = 1,221 (M: 627; F: 594) aged 65 years and older	Up to 70% of elderly individuals in the United States consume less than 1.00 g of creatine daily, with approximately 19.8% consuming no creatine at all. Those with suboptimal creatine intake were found to have a 2.62-fold increased risk of angina pectoris and a 2.59-fold increased risk of liver conditions compared to their counterparts who consume 1.00 g or more of creatine per day, after adjusting for demographic and nutritional variables	Ostojic et al. (2021)
NHANES 2017–2018. *n* = 4004 (F: 51.7%) aged 51.6 [17.8] years	Almost two-thirds of US adults consume dietary creatine below recommended levels of 1.0 g per day	Ostojic (2021)
NHANES 2013–2014. *n* = 1912 (F: 52.2%) aged 20–75 years	A significant inverse correlation was observed between dietary creatine intake and serum levels of neurofilament light chain (NfL), a recognized biomarker for neuronal damage. This finding indicates that creatine may exert protective effects against neuronal injury	Ostojic et al. (2024)
NHANES 2017–2020. *n* = 4522 females aged 44.5 [20.5] years	A strong correlation was observed between increased dietary creatine intake and a reduced risk of oligomenorrhea. A creatine-rich diet has been associated with decreased risks of reproductive health issues among U.S. women. Specifically, females consuming ≥13 mg of creatine per kilogram of body weight per day exhibited significantly lower risks of experiencing irregular menstrual cycles, obstetric complications, and pelvic pathologies	Ostojic et al. (2024)
Meta-analytic clinical evidence		
*n* = 357 both men and women aged 63.6 [5.9] years	The results from this meta-analysis are encouraging in supporting a role for creatine monohydrate supplementation during resistance training in healthy aging by enhancing muscle mass gain, strength, and functional performance over resistance training alone	Devries & Phillips (2014)
*n* = 721 both men and women aged 57–70 years	Creatine supplementation during resistance training results in ∼1.4 kg greater increase in lean tissue mass when compared to placebo. This translates to significantly greater increases in upper body (chest press) and lower body (leg press) strength in older adults	Chilibeck et al. (2017)
*n* = 583 both men and women aged 48–77 years	This systematic review concluded that creatine monohydrate is safe to use in older adults. Also, in conjunction with moderate- to high-intensity exercise, creatine supplementation may improve skeletal muscle health in the aging population	Stares & Bains (2019)
*n* = 50 (mainly men) aged >65 years	This systematic review and meta-analysis aimed to summarize these factors influencing the efficacy of nutritional interventions on muscle mass in older adults. Pooled summary effect indicated that creatine monohydrate supplementation is an effective intervention for increasing muscle mass measures in older adults	Martin-Cantero et al. (2021)
*n* = 60 both men and women aged >60 years	In the subgroup analysis of the types of nutrients, only creatine showed synergistic effects with resistance training on lean body mass	Choi et al. (2021)
*Lean tissue mass n* = 509 both men and women aged >50 years *Muscle strength n* = 426 both men and women aged >50 years	Regardless of dosing strategy, creatine monohydrate enhances lean tissue mass and strength gains from resistance training compared to placebo. Subanalyses revealed that a creatine-loading phase followed by maintenance dosing (≤5 g/day) significantly increased chest press strength. Higher doses (>5 g/day), both with and without loading, resulted in substantial gains in leg press strength. Additionally, supplementation on training days alone significantly improved lean tissue mass and strength compared to placebo	Forbes et al. (2021)
*n* = 176 women aged 56–70 years	Creatine monohydrate plus resistance training in a small cohort of older females enhanced muscle strength when the duration was at least 24 weeks; however, there was no effect on muscle mass	Dos Santos et al. (2021)
8 systematic reviews with meta-analysis. *n* = 784 both men and women aged ≥65 years	In this umbrella review of systematic reviews and meta-analyses, the authors concluded that data suggest a positive effect of creatine supplementation on top of progressive resistance training on muscle mass and muscle strength	Gielen et al. (2021)
*n* = 1,192 both men and women	Adding creatine supplementation to a resistance training program results in an increase in lean body mass. In adults over 48 years old, creatine supplementation alone (with or without exercise) increased lean mass by ∼0.6 kg. When combined with resistance training, the increase in lean body mass was greater, with an additional gain of 0.47 kg, totaling ∼1.1 kg	Delpino et al. (2022)
*n* = 225 (M: 74, F: 151)	Creatine monohydrate supplementation enhances measures of memory performance in healthy individuals, especially in older adults (66–76 years)	Prokopodis et al. (2023)
*n* = 492 both men and women aged 20–76 years	Recent evidence indicates that creatine monohydrate supplementation may have positive effects on cognitive function in adults, particularly in areas such as memory, attention span, and information processing speed	Xu et al. (2024)
*n* = 1,076 both men and women aged 42–72 years	Creatine supplementation enhances sit-to-stand performance, muscle function, and lean tissue mass in individuals at risk of functional disability	Davies et al. (2024)
Regulatory Agencies		
Food and Drug Administration (FDA) – United States of America	The FDA expressed no objections to a Generally Recognized as Safe (GRAS) application by Alzchem Group AG, permitting the inclusion of creatine monohydrate as a food additive in various food products (GRAS Notice 931) “*Based on the totality of data and information presented in the notice, Alzchem concludes that creatine monohydrate is GRAS for its intended use in food*”	GRAS Notice Inventory – GRN No. 000931 (USFaD and Inventories, 2020)
European Food Safety Authority (EFSA) – European Union	The EFSA panel concluded that a cause-and-effect relationship has been established between the consumption of creatine in combination with resistance training and improvement in muscle strength in adults over the age of 55 “*In order to obtain the claimed effect, 3 g of creatine should be consumed daily in conjunction with resistance training, which allows an increase in the workload over time. Resistance training should be performed at least three times per week for several weeks, at an intensity of at least 65%–75% of one repetition maximum. The target population is adults over the age of 55 who are engaged in regular resistance training*.”	EFSA NDA Panel (2016) (EFSA Panel on Dietetic Products NaAN, 2016)
Natural and Non-prescription Health Products Directorate (NNHPD) – Health Canada	Creatine monohydrate was assigned a monograph by the NNHPD that overviews research on this product to substantiate safety and efficacy. Only products containing creatine monohydrate can benefit from an abbreviated licensing process by referencing the monograph	NNHPD 28th June 2024 (Canada, 2024)
Ministry of Health, Labor and Welfare (MHLW) – Japan	Creatine monohydrate (クレアチン) is considered a “non-drug” that is allowed to be sold as a food ingredient and additive under the Food Sanitation Act (Act No. 46 of 2018). Health claims of creatine monohydrate for muscle maintenance with exercise was accepted in 2019	MHLW No. 0328 on 28th March 2024 (Ministry of Health LaW)
Spanish Agency for Food Safety and Nutrition (AESAN) – Spain	The Scientific Committee considers that the maximum daily amounts of 3.41 g of creatine monohydrate provides a maximum daily amount of 3 g/day of creatine and is acceptable from the standpoint of its safety in use as food supplements for a healthy adult population	AESAN Scientific Committee (2024) (Fernández et al., 2024)

The understanding of creatine’s potential cognitive benefits has advanced with recent studies, yet significant research is still needed to clarify the underlying mechanisms. Previously, we have described plausible biological regulators mediating effects of creatine supplementation using convergent functional genomics (Bonilla et al., 2021b) and have also found that cellular allostasis relies heavily on the creatine kinase/phosphocreatine system, which contributes to the intricate balance of subcellular energy production and cellular mechanics (Bonilla et al., 2021a). This reliance has been clinically evidenced by improvements in cerebral high-energy phosphates, cognitive performance, and processing speed, as reported by Gordji-Nejad et al. (2024) following a high single dose of creatine monohydrate (0.35 g/kg) during sleep deprivation. Furthermore, Bian et al. (2023) provided valuable insights into the potential role of creatine as a neurotransmitter, presenting strong evidence of creatine’s presence within synaptic vesicles. Although promising, these cognitive improvements require standardized measurement approaches and further investigation into their clinical implications for older adults at risk of neurodegenerative diseases. To address these gaps, we invite the scientific community to contribute their data and insights to our research topic “Exploring Creatine Supplementation: Enhancing Physical and Cognitive Health in Older Adults”, where the latest findings, next research directions, and collaborative efforts on creatine for health and disease will be collected. For example, in addition to what is listed in Table 1, more clinical research on creatine benefits in preventing the development of several other aging long terms conditions are needed (e.g., diabetes, cardiovascular disease, orthopaedical function, frailty, etc.).

**FIGURE 1:**
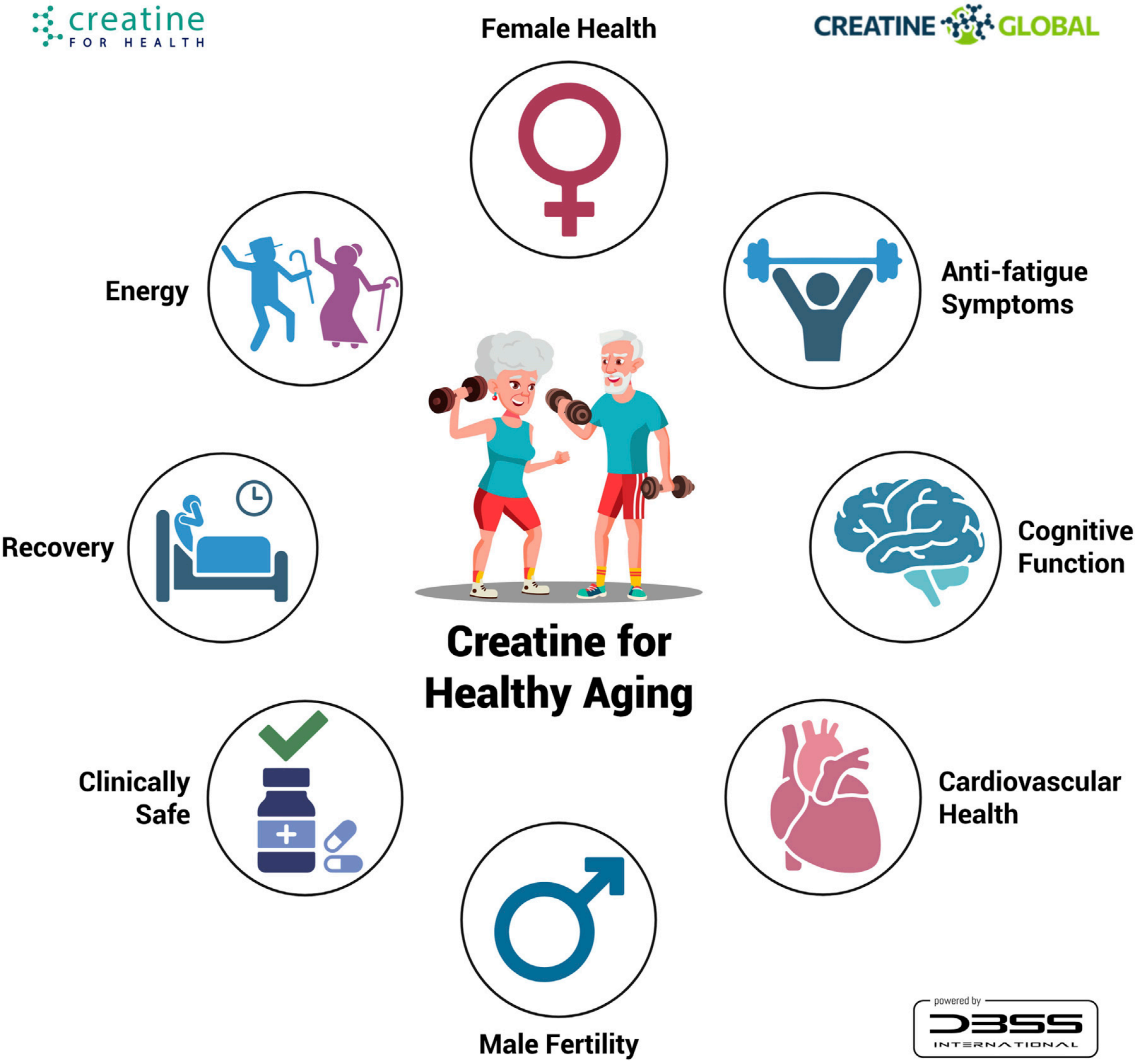
Benefits of creatine monohydrate supplementation plus resistance training in older adults.

Overall, creatine supplementation in combination with resistance training serves as a safe and effective approach to counteract the progression of sarcopenia (Figure 1). It is clear that creatine monohydrate supplementation—regardless of creatine loading, maintenance dosage, or frequency of ingestion—during a resistance training program increases lean tissue mass and strength compared to a placebo and resistance training alone in older adults (Forbes et al., 2021b). We advocate for the prompt implementation of public health initiatives that promote the inclusion of creatine-rich foods in human nutrition (www.creatine.global) and invite practitioners to visit www.creatineforhealth.com, an initiative that brings together creatine researchers worldwide to accelerate awareness about the role of creatine supplementation for health and in clinical diseases.
